# Stackable Medical-Grade Skincare for the Cosmetic Medicine Patient: A Long-Term Pilot Assessment

**DOI:** 10.1093/asjof/ojae037

**Published:** 2024-06-05

**Authors:** Sydney Pryor, Alec Semersky, Tiffany Sabev, Julius Few

## Abstract

**Background:**

Multiple intrinsic and extrinsic factors influence aging and lead to visible changes in the skin, including dryness, fine lines and wrinkles, loss of elasticity, surface roughness, uneven pigmentation, and loss of luminosity. Although it is well established that a single combination of topicals can address multiple signs of skin aging, it is common for patients’ at-home skin treatment routines to include multiple different topicals with different active ingredients. The layering of active ingredients can cause skin irritation, and lead to noncompliance with a consistent routine. Further, multiple active ingredients may exacerbate irritation from in-office aesthetic treatments.

**Objectives:**

To assess the long-term efficacy, safety, tolerability, and patient adherence to a Stackable Treatment topical routine consisting of 4 complementary topical formulations.

**Methods:**

This study examined a daily topical routine (Stackable Treatment routine) consisting of 4 topical formulas with different active ingredients, and evaluated the routine's safety, tolerability, and efficacy in a composite of in-office treatment patients who applied the routine for a minimum of 1 year.

**Results:**

Of the 14 patients, 0 experienced adverse reactions. Improvements to multiple skin parameters were observed, including improvements to skin hydration, surface texture, pigmentation, vasculature, and the appearance of scars. The majority of patients continue to use the Stackable Treatment routine after the study's conclusion.

**Conclusions:**

The combination of low incidence of irritation, high patient satisfaction, and overall efficacy of the routine indicates the Stackable Treatment routine may be well suited as a foundational skin care regimen that can complement in-office aesthetic treatments.

**Level of Evidence: 4:**

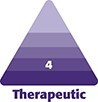

Both intrinsic and extrinsic factors, and interactions between them, contribute to skin aging. Intrinsic factors include collagen and elastin loss over time, genetic predisposition, and hormonal changes, whereas extrinsic factors include diet, environmental conditions, pollutants, chemical exposure, and ultraviolet light exposure.^1^ Intrinsic aging within the skin is caused by decreased capacity of keratinocytes, fibroblasts, and melanocytes to proliferate and the degeneration of the fibrous extracellular matrix, whereas up to 80% of extrinsic aging can be attributed to ultraviolet (UV) radiation exposure.^2-5^ For intrinsic and extrinsic factors, reactive oxygen species (ROS) and oxidative stress accelerate the processes of aging, and contribute to dyspigmentation, loss of elasticity, reduced barrier function, uneven texture, and fine lines and wrinkles.^1,6^

As intrinsic and extrinsic factors manifest into multiple different visible signs of aging, such as dyspigmentation, sun damage, laxity, fine lines, and wrinkles, patients look for multiple treatment modalities to improve their appearance. In the senior author's clinical practice, stacking therapies, or using multiple nonsurgical therapies in a single setting to approach a surgical result, were introduced by the lead author in 2012.^7-9^ This now-common approach involves combining absorbable suture suspension with other modalities (eg, energy-based therapies [microfocused ultrasound and laser]), as well as neuromodulator, filler, and fat transfer, in the same setting.^7,8^ The stacking of nonsurgical therapies effectively addresses multiple signs of aging, such as volume loss, skin sagging, uneven skin, wrinkles and fine lines, and brow ptosis, through different modalities.^7-9^

The effectiveness of stacking nonsurgical therapies is well established; however, the same stackable approach has not been thoroughly described in the literature for the stacking of topical skin treatments. In the lead author's clinical practice, both a combination retinol, peptide, cannabidiol topical and a topical serum combining postbiotics, peptides, and botanical extracts demonstrated clinical benefit in addressing different skin health and aging parameters.^5,10^ However, though effective, both of these combination topicals provide only a part of what can be referred to as a comprehensive skincare routine. In the author's experience, weaknesses in skincare include inadequate sun protection; the use of too-aggressive products that cause irritation, dryness, or excess sebum production; the use of too many products that do not adequately or specifically support skin health; the lack of adherence to a consistent routine; and the use of products that contain irritants, perfumes, or colorants that could worsen the patients’ skin and may have negative impacts on overall health.^10^ In the current retrospective study, a routine of 4 different topicals applied daily (Stackable Treatments, Aforé LLC, Chicago, IL) was evaluated that included a glycolic acid foaming cleanser; a sun protection factor (SPF) 30 mineral (25% zinc oxide) sunscreen; a combination emollient/occlusive/humectant moisturizer; and a combination retinol/peptide topical with retinol strength varying between patients between 0.2%, 0.5%, and 1%. By stacking these 4 topicals daily, in a specific order, the Stackable Treatment routine was designed to combat both intrinsic and extrinsic factors involved in skin aging by removing impurities and pollutants from the skin, preserving skin barrier function, preventing skin moisture loss, and protecting the skin through antioxidative and antiinflammatory mechanisms such that efficacy and tolerability were improved.

In this retrospective study, because of the patient pool, including patients who underwent both nonsurgical and surgical procedures, a more granular approach was taken toward measuring outcomes. Typically, aesthetic outcomes would be measured using validated and nonvalidated scales, and using static photographs taken from multiple angles.^11,12^ Alternatively, as the lead author has done in 2 previous studies on combination topicals, a 9-domain Global Ranking Scale and 4-domain Skin Quality Assessment could be performed.^5,10,13^ However, in the case of this retrospective study, a binary questionnaire (yes showed improvement, no improvement shown, or not applicable) was used to evaluate improvements to different aesthetic skin parameters. The domains selected were chosen because they are common in-office skin quality concerns and were reported by the majority of patients included in the study. Additionally, it was important to evaluate patient adherence to the Stackable Treatment routine over a relatively long time period during which patients were also undergoing both surgical and nonsurgical aesthetic treatments. Reviewing patients’ electronic medical records (EMRs) for continued adherence, in addition to all reports of adverse skin reactions, was performed. For the purposes of this study, long-term patient satisfaction, adherence to the routine, minimization of adverse reaction, and minimization of irritation were the markers of success. Establishing a long-term effective, demonstrably adherable Stackable Treatment routine that patients adhere to even after in-office procedures will create the foundation for further clinical study.

## METHODS

### Study Design

This single-center retrospective pilot study assessed the efficacy, tolerability, and adherence to a Stackable Treatment routine consisting of 4 topical products. The routine consisted of a glycolic acid foaming cleanser used once per day to remove environmental pollutants, dead skin, and excess oil and sebum; a combination emollient/occlusive/humectant moisturizer used twice per day to maintain skin barrier function and prevent water loss; an SPF 30 mineral (25% zinc oxide) sunscreen used once per day in the morning to reduce UV-induced skin damage; and a combination retinol and peptide topical with retinol strengths varying between 0.2%, 0.5%, and 1%. An ingredient list as well as application instructions is summarized in Table 1 for each of the Stackable Treatment topical formulations. The inclusion criteria are 18 years of age and older, existing patients at the senior author's clinic, and the presence of complete medical records between January 1, 2021 and December 31, 2023.

**Table 1.** Stackable Treatment Ingredients and Application Instructions

Topical	Key ingredients	Application instructions
Glycolic acid cleanser	Glycolic acid	Squeeze the product into wet hands. Work into lather and smooth over moistened face using gentle, circular motions. Rinse off with lukewarm water. Pat dry. Follow with the application of a combination moisturizer
Combination moisturizer	Green tea extract Sunflower seed oil Babassu oil Olive oil Coconut oil	Apply 2-3 pumps of product to the fingertips. Massage cream into facial skin morning and evening
Sunscreen	Active ingredient: 25% zinc oxide Inactive ingredients: jojoba oil	Apply a liberal amount of sunscreen to your fingertips. Spread product evenly to all areas of the face, neck, and décolletage each morning after cleansing the skin with glycolic acid cleanser and applying a combination moisturizer Reapply sunscreen every 90 min, after swimming, and after towel drying for optimal sun protection
Retinol + peptide topical (varying retinol concentrations between 0.2%, 0.5%, and 1%)	Retinol Green tea extract Matrixyl Argireline Olive oil Coconut oil Babassu oil	In the evening before bed, apply 2-3 pumps to clean fingertips. Apply cream using fingertips to freshly cleansed face, neck, and décolletage area until absorbed

A total of 14 patients’ EMRs were reviewed; demographics are summarized in Table 2. Patients voicing concerns about skin quality related to aging, who simultaneously had been using the Stackable Treatment routine as prescribed by the senior author, were included. Patients who had received botulinum toxin A, filler, energy-based treatments, or rejuvenation procedures to the face or neck (eg, microneedling, microdermabrasion, and chemical peels) were included in the study. A breakdown of which surgical and nonsurgical procedures were performed during the retrospective study duration is summarized in Table 3. Those who had uncontrolled systemic inflammatory conditions, had active dermatologic conditions in the face or neck, had known allergies to the ingredients, were pregnant, used other skin care, and had an inability to perform skin care were excluded.

**Table 2.** Study Demographics

Median age, years (range)	60.8 (36-74)
Gender, *n* (%)	
Male	0 (0)
Female	14 (100)
Fitzpatrick skin type, *n* (%)	
I	0 (0)
II	5 (35.7)
III	7 (50)
IV	1 (7.1)
V	0 (0)
VI	1 (7.1)

**Table 3.** Aesthetic Questionnaire Results

Parameter	Skin roughness	Dehydration	Scars	Visible pores	Pigmentation	Vasculature
# Respondents	14	14	14	14	14	14
Showed improvement	13	13	9	12	13	8
No improvement	0	0	0	0	1	1
Not applicable	1	1	5	2	0	5
% of applicable Patients demonstrating improvement	100	100	100	100	92.9	88.9

Patients were called individually and asked a series of questions on the Stackable Treatment protocol within 4 weeks of the end of the course of their treatment. Each was asked the following questions: (1) Which topical products (if any) are you continuing to use today? (2) Did you experience improvement in skin roughness while using Stackable Treatments? (3) Did you experience improvement in skin dehydration while using Stackable Treatments? (4) Did you experience improvement in the appearance of scars while using Stackable Treatments? (5) Did you experience improvement in the appearance of visible pores while using Stackable Treatments? (6) Did you experience improvement in the pigmentation while using Stackable Treatments? (7) Did you experience improvement in vasculature while using Stackable Treatments? Patients were called by a licensed medical aesthetician with extensive experience with prospective clinical studies, who additionally evaluated before and after photographs for visible improvements. Patient answers to the phone call survey were recorded and tabulated.

In this retrospective study, there was no placebo group. Compliance was assured by reviewing patient repurchase frequency, EMR assessment, and verbal patient confirmation over the phone during the follow-up questionnaire. With perfect use, topical products included in this study lasted 60 days. Patients with a gap greater than 60 days between product purchases were not considered compliant and were not included in this study. The licensed aesthetician conducting phone calls asked patients 2 questions to ensure compliance: (1) What is your daily skincare routine in the morning and evening? (2) Do you use the glycolic cleanser, combination moisturizer, zinc oxide sunscreen, and retinol daily? If patients did not respond that they used all 4 topicals daily, they were not considered eligible for the study.

Adverse reactions, skin irritation, and any patient dissatisfaction were recorded in patients’ EMRs, and all provider notes were reviewed from pre and post in-clinic skin procedure consultations (Hydrafacial, Dermaplane, etc). Follow-up was confirmed by an in-person assessment and/or phone call.

This study adhered to the standards of the World Medical Association's 1975 Declaration of Helsinki, including the revisions of 2000 and 2008. Consents for the photographs included in this study were obtained.

## RESULTS

A total of 14 patients used the Stackable Treatment skincare routine daily between February 2021 and November 2023. There were 14 female patients and 0 male patients. The median age was 62 years (range, 32-74 years). The patient demographics are summarized in Table 2. The study follow-up period was a minimum of 1 year (range, 374-967 days; average follow-up period, 606 days; median follow-up period, 546 days).

Out of the 14 patients, 14 (100%) responded to the aesthetic questionnaire. The results from the questionnaire are as follows: all 13 (100%) patients presenting with skin roughness demonstrated improvement to skin roughness; all 13 (100%) patients presenting with skin dehydration demonstrated improvement to skin dehydration; all 9 (100%) patients presenting with visible scars demonstrated improvement to the appearance of scars; all 12 (100%) of patients presenting with visible pores demonstrated improvement to the appearance of visible pores; 13 of the 14 (92.9%) patients presenting with pigmentation demonstrated improvement to pigmentation; and 8 of the 9 (88.9%) patients presenting with vasculature demonstrated improvement. The results are summarized in Table 3.

Of the 14 patients included in this study, none experienced adverse skin reactions or skin irritation during the study duration.

Of the 14 patients, 14 (100%) received nonsurgical treatment during the study duration, and 2 (12.5%) patients received surgical treatment during the study duration. The treatments performed during the study duration are summarized in Table 4.

**Table 4.** In-Office Aesthetic Procedures

Procedure	Patients receiving during study period, *n* (%)
Dermaplaning	12 (57.1%)
Hydrafacial	14 (66.6%)
HALO laser	5 (23.8%)
Laser resurfacing	1 (4.8%)
Sofwave	5 (23.8%)
Silhouette lift	3 (14.3%)
Restylane	1 (4.8%)
Dysport	12 (57.1%)
VI peel	7 (33.3%)
Ulthera	7 (33.3%)
Fillers	7 (33.3%)
Facelift	1 (4.8%)
Skin pen	3 (14.3%)
Lip lift	1 (4.8%)
Pelleve	1 (4.8%)
Venus freeze	2 (9.5%)
Moxi	2 (9.5%)
Broadband light laser	7 (33.3%)
Blepharoplasty	1 (4.8%)

Patients continued to use the Stackable Treatment topicals after the study concluded. All the 14 (100%) study patients responded with their current skincare routines, and they continued to use the retinol topical. Nine of the 12 respondents (75%) continued to use physical sunscreen. Eleven of the 14 (78.6%) respondents continued to use the glycolic cleanser. Thirteen of the 14 (92.9%) continued to use the combination moisturizer.

## DISCUSSION

**Figure 1.**
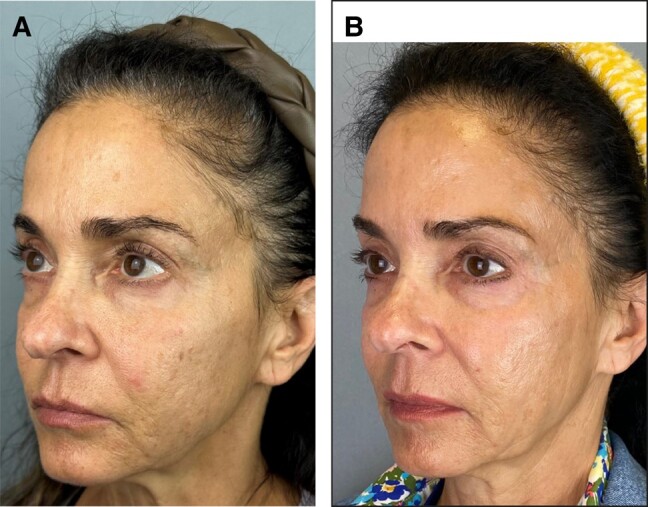
(A) A 65-year-old female patient at baseline with facial skin characterized by rough skin texture, hyperpigmentation, and dehydration. (B) Patient after 922 days of daily application of Stackable Treatment topicals. Note the improvements to skin texture and hyperpigmentation, as well as the improvement to skin hydration indicated by the more even distribution of light.

**Figure 2.**
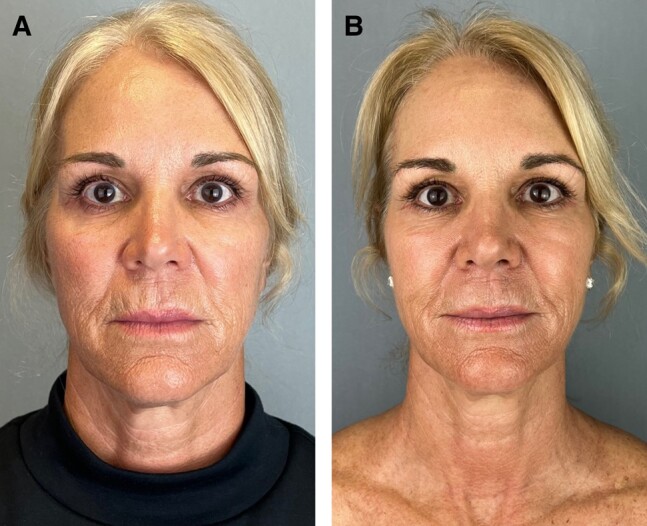
(A) A 64-year-old female at baseline with facial skin characterized fine lines and wrinkles and rough skin texture. (B) Patient after 487 days of daily application of Stackable Treatment topicals. Note the significant decrease in the appearance of fine lines and wrinkles, as well as the improvements to skin texture.

Stackable Treatments, as described in this retrospective study, are a novel way of prescribing a long-term, comprehensive skincare routine that includes ingredients known to support several aspects of skin health. To our knowledge, this is the first study to review both the long-term efficacy and tolerability of a multitopical skin care routine over multiple years—notably, a routine that is used concomitantly with in-office surgical and nonsurgical aesthetic procedures. This study provides evidence that multiple topical products can be used effectively and over a relatively long time period (median follow-up, 494 days) to comprehensively address intrinsic and extrinsic factors that contribute to skin health and aging. Evaluation of patients before and after (Figures 1, 2), as well as questionnaires with the patients, shows an improvement to skin surface roughness, dehydration, scars, pigmentation, visible pores, and vasculature while using the Stackable Treatments topical routine. Alongside this improvement, both the evaluation of patients’ EMRs and the questionnaire demonstrate a remarkably low incidence of irritation while using the Stackable Treatment routine. No irritation was reported during the study, demonstrating the long-term tolerability of the routine. The combination of the routine's efficacy, long-term adherence, and tolerability suggests the Stackable Treatment routine is well suited for foundational skincare, inclusive of a wide array of other in-office procedures they may be receiving.

The effects of natural extracts on the skin can be difficult to predict, especially when used in combination, layered on top of one another, or used in a daily routine. As the Stackable Treatment routine employs natural botanical extracts; in addition to laboratory-made ingredients, pinpointing exact mechanisms of action between each product is beyond the scope of this paper. Clinical investigation is critical for defining efficacy and tolerability. In lieu of a full literature review and investigation into mechanisms of action, the rationale behind the main active ingredients in each topical is given below.

In clinical practice, retinol is one of the few topical ingredients for which there is a significant body of evidence supporting its efficacy.^5,14^ Retinol's efficacy can be attributed to its ability to promote the proliferation of keratinocytes, which strengthens the epidermis, and increases collagen production. However, retinol can leave skin prone to damage from ROS, as it can reduce skin's antioxidative capacity,^5,15-17^ This, in addition to retinol's association with redness, peeling, flaking, and sun sensitivity, can lead to inconsistent application, ceasing of application altogether, or patient dissatisfaction. Thus, although retinol has been thoroughly demonstrated in the literature to provide benefits to the skin's appearance and health, its potential for irritation by itself can risk patients’ consistency in application. This irritation risk, and potential for ceasing daily application, may be more pronounced when retinol is layered with other active ingredients. However, this study included patients using a retinol/peptide combination topical of varying strengths between 0.2%, 0.5%, and 1%. Retinol strengths were chosen based on each patient's individual aesthetic needs as determined by their provided information, in combination with the patient's self-reported retinol tolerability. Notably, neither irritation nor adverse reactions were reported, even among the patients using the highest retinol strength (1% concentration). This can be partially attributed to the retinol's formulation, which was inclusive of both peptides and botanical extracts that may help to reduce oxidative stress and provide an antiinflammatory skin benefit. It is also likely attributable to the other 3 Stackable Treatment topicals utilizing ingredients that protect the skin through antioxidative and inflammatory mechanisms, so that both efficacy and tolerability of all of the topicals, including the retinol, were improved.

Green tea was incorporated into the combination moisturizer for its high percentage of polyphenols and antioxidant capacity, specifically Epigallocatechin gallate (ECGC). ECGC is one of the main constituents of green tea and has been demonstrated to exhibit high antioxidant capacity and skin-soothing benefits.^18,19^ To further decrease the potential for irritation and dryness caused by retinol, in-office procedures including chemical peels, UV exposure, and environmental factors, a combination moisturizer was chosen. The moisturizer contained a combination of 4 botanical-based oils. Babassu oil, sunflower seed oil, and olive oil contain compounds with antimicrobial, antiitch, antiinflammatory, and antioxidant properties.^20-22^ Environmental factors, pollution, aesthetic treatments, and UV exposure can each affect skin barrier function through different independent mechanisms. Jojoba oil—incorporated into the topical sunscreen—and sunflower seed oil each contain fatty acids and nutrients shown to improve skin hydration and preserve stratum corneum integrity with a low risk of irritation.^20,21^ In order to preserve the skin barrier—specifically to prevent moisture loss—the combination moisturizer included an occlusive component of coconut oil. Coconut oil has been demonstrated to reduce transepidermal water loss and help preserve skin barrier function in patients with atopic dermatitis.^23^

To protect the skin from UV radiation exposure, a mineral SPF was selected as the daily topical for Stackable Treatments. This topical included a 25% zinc oxide component providing SPF 30 protection, which blocks up to 97% of UV exposure. Zinc oxide is an FDA-approved topical sunscreen ingredient for which there is a significant body of evidence supporting its efficacy in reducing sun damage.^24^

Morning and evening, patients using the Stackable Treatment routine utilized a glycolic acid–based foaming cleanser with lukewarm water. While on a retinol regimen, skin cell turnover is increased, which leads to an increase in dead skin cells on the outer layer of skin. This accumulation of dead skin cells can hinder the absorption of topical formulas; thus, to improve the absorption of the other Stackable Treatment topicals, the removal of dead skin cells is an important step. To address this accumulation, glycolic acid was chosen as an exfoliating agent in the cleanser. Topically, glycolic acid in the form of chemical peels has been shown to reduce wrinkle depth and appearance.^25^ However, chemical peels can lead to irritation and skin sensitivity, especially when left on the skin for a prolonged period.^25^ When incorporated into a cleanser, however, glycolic acid can provide a relatively gentle exfoliation that removes dead skin cells, environmental pollutants, and oil without irritating scrubbing action or sitting on top of the skin for an extended period. In this way, and with a once- or twice-daily application, glycolic acid can provide the benefits of dead skin cells and pollutant removal.

The strengths of this study are several. The long-term evaluation of efficacy, safety, and tolerability for over 1 year of multiple complementary formulations is unique to this study. This evaluation included a wide range of patient skin types, suggesting the Stackable Treatment routine is effective and tolerable across different skin tones and types. To further emphasize efficacy and patient satisfaction, the topical products were purchased by patients in the study, suggesting both satisfaction and a willingness to continue using the products. Additionally, it is notable that all 14 patients (100%) underwent nonsurgical and surgical procedures during the retrospective analysis period and continued to use the Stackable Treatment routine daily. This preliminary finding suggests the Stackable Treatment routine can continue to be used after in-office aesthetic rejuvenation procedures without causing additional irritation and may suggest the routine can help reduce irritation caused by in-office procedures. This suggests the Stackable Treatment routine may complement in-office procedures, and further improve skin health and appearance. This suggests the Stackable Treatment routine may complement in-office procedures, and further improve skin health and appearance in a protocol that is easy and comfortable for patients to follow at home. Clinical confirmation will require further, controlled study.

Shortcomings of this study include the absence of a control group, confounding variables such as patients undergoing in-office aesthetic procedures during the study, and the absence of a validated scale to measure changes to the skin. Patients having undergone various surgical rejuvenation procedures during the study create complications in evaluating before and after results, and discerning aesthetic differences attributable specifically to topical Stackable Treatment topicals. In future study, the analysis of Stackable Treatments would benefit from a prospective design, rather than a retrospective design, inclusive of a control arm without in-office procedures, as well as a validated scale for skin assessment. This study did not include patients of each Fitzpatrick skin type, nor any male patients. A future study would also benefit from a larger and more diverse cohort, inclusive of all Fitzpatrick skin types, as well as male patients.

Stackable Treatments were designed as a simplified, gentle, effective skincare routine with a low incidence of irritation and long-term adherence, even while patients undergo other nonsurgical and surgical aesthetic procedures. The routine was designed to address intrinsic and extrinsic factors that contribute to skin aging, and address the shortcomings of other skincare formulations and routines as seen by the lead author in his clinical practice. This study supports a role for the formulation of complementary skincare formulas that address a wide array of skincare concerns in a way that limits irritation, and simultaneously addressing the multiple intrinsic and extrinsic factors behind aging skin and skin health.

## CONCLUSIONS

Based on this retrospective study, the Stackable Treatment routine, consisting of 4 topical formulations appears to be an effective and well-tolerated daily topical routine designed to address an array of skin quality and skin health issues. In particular, the Stackable Treatment routine demonstrated improvement with respect to skin roughness, dehydration, pigmentation, scar appearance, visible pore size, and vasculature. The Stackable Treatment routine demonstrates high patient adherence, and no incidence of irritation when used concomitantly with a wide range of in-office nonsurgical and surgical procedures, suggesting it is an effective, simple multitopical routine that can be used by various skin tones and skin types without causing irritation at the same time as in-office aesthetic rejuvenation procedures.
